# Circulatory Mitochondrial DNA Is a Pro-Inflammatory Agent in Maintenance Hemodialysis Patients

**DOI:** 10.1371/journal.pone.0113179

**Published:** 2014-12-08

**Authors:** Hongdi Cao, Hong Ye, Zhiping Sun, Xia Shen, Zongwei Song, Xiaochun Wu, Weichun He, Chunsun Dai, Junwei Yang

**Affiliations:** Center of Kidney Disease, 2nd Affiliated Hospital, Nanjing Medical University, Nanjing, Jiangsu Province, China; University of Sao Paulo Medical School, Brazil

## Abstract

Chronic inflammation is highly prevalent in maintenance hemodialysis (MHD) patients, and it has been shown to be a strong predictor of morbidity and mortality. Mitochondrial DNA (mtDNA) released into circulation after cell damage can promote inflammation in patients and animal models. However, the role and mechanisms of circulatory mtDNA in chronic inflammation in MHD patients remain unknown. Sixty MHD patients and 20 health controls were enrolled in this study. The circulatory mtDNA was detected by quantitative real-time PCR assay. Plasma interleukin 6 (IL-6) and tumor necrosis factor α (TNF-α) were quantitated by ELISA assay. Dialysis systems in MHD patients and in vitro were used to evaluate the effect of different dialysis patterns on circulatory mtDNA. Circulatory mtDNA was elevated in MHD patients comparing to that of health control. Regression analysis demonstrated that plasma mtDNA was positively associated with TNF-α and the product of serum calcium and phosphorus, while negatively associated with hemoglobin and serum albumin in MHD patients. MtDNA induced the secretion of IL-6 and TNF-α in the THP-1 cells. Single high-flux hemodialysis (HF-HD) and on line hemodiafiltration (OL-HDF) but not low-flux hemodialysis (LF-HD) could partially reduce plasma mtDNA in MHD patients. In vitro, both HD and hemofiltration (HF) could fractional remove mtDNA. Collectively, circulatory mtDNA is elevated and its level is closely correlated with chronic inflammation in MHD patients. HF-HD and HDF can partially reduce circulatory mtDNA in MHD patients.

## Introduction

Chronic inflammation is one of the characteristics of maintenance hemodialysis (MHD) patients, which has been shown to be a strong predictor of morbidity and mortality in this population [1]. It was demonstrated that chronic inflammation played an important role in the pathogenesis of cardiovascular disease (CVD) in MHD patients [2]. The increased levels of specific cytokines (interleukin 6 and tumor necrosis factor α) or acute phase proteins (C-reactive protein) have been found to be associated with CVD in MHD patients [3], [4]. Inflammation in MHD patients may be related to processes associated with renal failure itself such as uremic toxins retention, oxidative stress and dialysis processes such as dialysis membranes and dialysis fluid [5]–[8]. However, the mechanism of chronic inflammation state has not been clarified clearly.

Containing the proinflammatory CpG DNA repeats is an improtant strcture feature for mitochondrial DNA(mtDNA) [9]. The proinflammatory role of mtDNA has been confirmed in several diseases such as severe trauma [10]. MtDNA escaped from autophagy in the cardiomyocytes by external hemodynamic stress could lead to Toll Like Receptor (TLR)-9 mediated inflammatory responses and is capable of inducing myocarditis [11]. Plasma DNA is also elevated in the septic patients, which has potential use for predicting outcome in septic patients arriving at the emergency room [12]. Post-dialysis cell-free DNA (cf-DNA) level might be an independent predictor of all-cause mortality in MHD patients [13]. It has been described that apoptotic cf-DNA was increased in the plasma of MHD patients, which selectively stimulated the production of pro-inflammatory cytokines from human monocyte [14]. Thus far, the change of circulatory mtDNA in MHD patients and the mechanism in the formation of inflammatory state has not yet be concerned.

In this study, the expression of circulatory mtDNA and several inflammatory markers were determined in MHD patients. The association among plasma mtDNA, inflammatory cytokines and clinical characteristics of patients was analyzed. To clarify the removal effect of different hemodialysis patterns on circulatory mtDNA, the clearance was evaluated in MHD patients and in vitro respectively.

## Materials and Methods

### Ethics statement

All of the following details were approved by the responsible ethics committee of Nanjing Medical University before the study. The Permit Number of this study was KY026. All patients and healthy controls provided written informed consent before any study-related procedure was performed.

### Study population

From Oct 2012 to Jan 2013, sixty MHD patients from the Center of Kidney Disease of 2^nd^ Affiliated Hospital of Nanjing Medical University and 20 healthy controls from the Medical Examination Center of the hospital were recruited. MHD was defined as patients undergoing regular hemodialysis prescription, three times a week, for 4 h per session at least 6 months. All patients were in a stable condition and free from infection for at least 3 months. Patients were divided into three groups according to the dialysis patterns: Group A: low-flux hemodialysis (LF-HD) with PMMA membrane (B3-1.3A, TORAY, JAPAN); Group B: high-flux hemodialysis (HF-HD) with polysulfone membrane (TS-1.3A, TORAY, JAPAN); Group C: on line hemodiafiltration (OL-HDF) with polysulfone filter (TS-1.3U, TORAY, JAPAN). All these groups with a blood rate flow of 250 to 280 ml/min and a dialysis rate flow of 500 ml/min and HDF with an additional dosage of substitution 30L. Each group of 20 patients received the treatment as indicated.

### Blood sampling

Two blood samples were collected for each patient: one before a single hemodialysis another at the end of it. Peripheral blood (2 ml) was collected into EDTA-containing tubes in each puncture. To ensure cell-free plasma collection, EDTA–blood were initially centrifuged for 10 min at 3000 rpm, followed by separation into a 1.5 ml clear polypropylene tube taking care not to disturb the buffer coat layer. The newly separated aliquot was centrifuged for a further 10 min at 10,000 rpm, after which the upper portion of plasma was removed and placed into a further clear tube and was frozen at −80°C prior to extraction [15]. DNA was extracted from 200 µl plasma samples with the use of a QIAamp DNA Mini and Blood Mini kit (Qiagen, Frederick, MD) using the “blood and body fluid protocol” as recommended by the manufacturer [16].

### Isolation of mitochondria and mtDNA preparation

Mitochondria of human skeletal muscle tissue which was from surgical trauma by traffic accident were isolated as described previously [17]. MtDNA was extracted from the isolated mitochondria using DNeasy Blood & Tissue kit (Qiagen, Frederick, MD), which was prepared under sterile conditions. The concentration of mtDNA was then determined spectrophotometrically. No protein contamination was found and nuclear DNA was less than 0.001% by quantitative polymerase chain reaction (PCR) [10]. The purified mtDNA was diluted into serial concentrations which were used as standards of real-time PCR protocols. Part of the purified mtDNA was used in the simulated dialysis systems.

### Real-time quantitative PCR protocols

Primers for human cytochrome B (Cyt B) (forward 5′-ATGACCCCAATACGCAAAAT-3′ and reverse 5′- CGAAGTTTCATCATGCGGAG-3′), human cytochrome C oxidase subunit III (COX III) (forward 5′-ATGACCCACCAATCACATGC-3′ and reverse 5′-ATCACATGGCTAGGCCGGAG-3′) were chosen for study because they are unique among mitochondrial molecules [18]. Primer sequences have no significant homology with DNA found in any bacterial species published on BLAST. Real-time PCR was performed using previously described primers. The PCR reaction mixture consisted of 1 ul of DNA, 12.5 µl of 2× SYBR Green PCR Master Mix (Roche Diagnostics GmbH Mannheim, Germany), 1 µl of forward and reverse primer, and 9.5 µl of nuclease-free water. PCR was carried out with an ABI Prism 7300 Sequence Detection System at 50°C for 2 min and 95°C for 10 min, followed by 40 cycles at 95°C for 15 s and 60°C for 1 min by amplifying serial dilutions of a known quantity of amplicons. Concentrations are expressed as nanograms per milliliter (ng/ml).

### Cell line culture, differentiation and stimulation

The role of mtDNA on inflammation was studied on the human acute monocytic leukemia cell line—THP-1 cell line, which was cultured as previously described [19]. Cells were maintained as a continuous culture in RPMI1640 medium supplemented with 10% fetal bovine serum (Invitrogen) in a humidified atmosphere of 5% CO_2_ in air at 37°C. The medium was refreshed every 3 to 4 days. Initially, the THP-1 cells (10^6^/ml) were differentiated toward the monocyte/macrophage phenotype in the presence of phorbol-12-myristate-13-acetate (PMA) 100 ng/ml for 36 hours. THP-1 cells were then incubated with purified mtDNA in different dosage from 0 hour to 32 hours to evaluate the effect on inflammatory markers.

### IL-6 and TNF-α ELISA

The amount of IL-6 and TNF-α in the supernatant of THP-1 cells and in the plasma of MHD patients and controls were determined by ELISA (R&D Systems, Wiesbaden, Germany) following the protocol recommended by the manufacturer.

### Simulated dialysis systems in vitro

**Figure 1:**
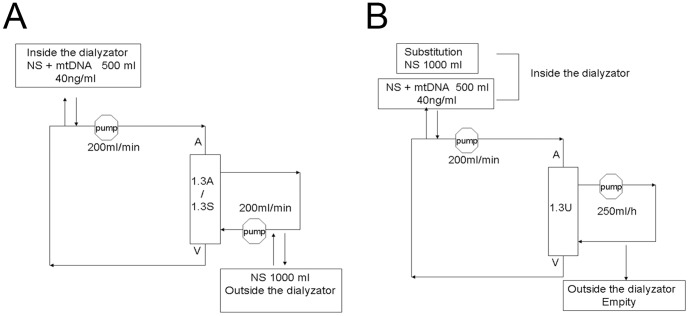
Three different dialysis systems, LF-HD, HF-HD and HF were established in vitro. A. The schematic diagram of LF-HD and HF-HD in vitro. B. The schematic diagram of HF in vitro.

Three simulated dialysis patterns, LF-HD, HF-HD and hemofiltration (HF) were established in vitro. The simulated dialysis system was shown schematically in Fig. 1. In LF-HD and HF-HD, saline 500 ml with 40 ng/ml mtDNA was used as blood and saline 1000 ml was used as dialysate (Fig. 1 A). For the HF system, saline 500 ml with 40 ng/ml mtDNA was used as blood and saline 1000 ml was used as substitution (Fig. 1 B). The samples 500 µl were taken from the blood and dialysate side after 4 hours for the detection of mtDNA. After completion of the simulated dialysis, the dialyzer and filter were soaked with saline 20 ml overnight followed by DNA extraction. Ten fibers were taken from each dialyzer and filter, incubated with tissue buffer lysis 5 ml at 37°C overnight.

### Statistic analysis

The data were analyzed using SPSS for windows version 18.0. Clinical data are expressed as the mean ± SD or median and range or frequency, as appropriate. Comparisons of continuous data of two groups used the independent *t* test. Compared *t* test was used in the data before and after hemodialysis. Logistic regression analysis was performed to describe the relationship between plasma mtDNA and clinical characteristics. A *P* value less than 0.05 was considered to represent a statistically significant difference.

## Results

### The clinical characteristics of MHD patients and controls

Baseline characteristics of participants were summarized in Table 1. MHD patients had a higher BMI (22.1±3.2 kg/m^2^) compared with controls (21.3±1.5 kg/m^2^, P = 0.001). Blood pressure especially systolic blood pressure (SBP) was higher in MHD patients (138.1±25.6 mmHg) than that in controls (106.5±10.6 mmHg, P = 0.005). The duration of hemodialysis was 57.2±45.6 months. MHD patients were more likely to have diabetes mellitus and the history of heart failure in recent one year. The hemoglobin (HGB) was lower in MHD patients than that in health controls, with 105.9±14.5 g/L vs 132.5±7.9 g/L (P = 0.034). Serum albumin (ALB) was 39.5±4.3 g/L in MHD patients, which was 46.9±3.1 g/L in controls. ALB adjusted Ca was 2.36±0.21 mmol/L in MHD patients and 2.28±0.13 mmol/L in controls (P = 0.060). Serum P and parathyroid hormone (PTH) were both higher in MHD patients than in controls, with serum P 1.71±0.55 mmol/L in MHD patients and 1.00±0.09 mmol/L in controls (P = 0.000); ln-PTH 4.60±1.38 ng/L in MHD patients and 3.57±0.38 ng/L in controls (P = 0.001). The product of Ca and P was also elevated in MHD patient.

**Table 1:** The clinical characteristics of MHD patients and health controls.

Clinical characteristics	Controls	MHD patients	*P* value
**No. of Cases**	N = 20	N = 60	-
**Age(years)**	41.0±11.7	57.7±11.3	0.685
**Male gender n (%)**	12(60.0)	34(56.7)	0.546
**BMI (kg/m2)**	**21.3±1.5**	**22.1±3.2**	**0.001**
**SBP (mmHg)**	**106.5±10.6**	**138.1±25.6**	**0.005**
**DBP (mmHg)**	69.3±8.3	79.3±11.8	0.127
**Duration of HD (months)**	-	57.2±45.6	-
**DM, n (%)**	**0(0)**	**9(15.0)**	**0.026**
**HF, n (%)**	0(0)	8(13.3)	0.091
**Smoking, n (%)**	6(30.0)	19(31.7)	0.386
**HGB (g/L)**	**132.5±7.9**	**105.9±14.5**	**0.034**
**Alb(g/L)**	46.9±3.1	39.5±4.3	0.209
**Ca(mmol/L)**	**2.40±0.11**	**2.35±0.20**	**0.012**
**Alb adjusted Ca (mmol/L)**	2.28±0.13	2.36±0.21	0.060
**Phosphate (mmol/L)**	**1.00±0.09**	**1.71±0.55**	**0.000**
**Product of Ca and P (mg/dl)^2^**	**27.3±2.8**	**48.6±16.8**	**0.000**
**Ln-Intact-PTH (ng/L)**	**3.57±0.38**	**4.60±1.38**	**0.001**
**CHOL (mmol/L)**	4.28±0.48	4.28±0.77	0.095
**LDL-C (mmol/L)**	2.25±0.10	2.31±0.60	0.636
**CRP (mg/L)**	**0.5–4.0**	**0.5–18.5**	**0.001**
**IL-6 (pg/ml)**	43.8±8.8	69.1±53.4	0.177
**TNF-α(pg/ml)**	**37.0±2.2**	**62.5±36.7**	**0.001**
**mtDNA (ng/ml)**	**42.7±8.5**	**129.1±101.3**	**0.000**

Abbreviations: BMI: body mass index; SBP: systolic blood pressure; DBP: diastolic blood pressure; DM: diabetes mellitus; HF: heart failure; HGB: hemoglobin; Alb: albumin; Ca: calcium; P: phosphorus; PTH: parathyroid hormone; CHOL: cholesterol; LDL-C: low density lipoprotein-cholesterol; CRP: C-reaction protein; IL-6: interleukin 6; TNF-α: tumor necrosis factor α.

### The circulatory mtDNA was significantly elevated in MHD patients

Circulatory mtDNA was evaluated from plasma of all 60 MHD patients before hemodialysis and 20 controls. Cyt B and COX III were applied to evaluate the quantity of mtDNA [18]. Cyt B was used as the main marker of mtDNA and COX III was used to validate the accuracy of Cyt B. It was shown that there was a strong correlation between the two mtDNA markers (Correlation coefficient 0.962, P = 0.000). In the 20 health controls, plasma mtDNA could be detected at a low concentration of 42.7±8.5 ng/ml, while plasma mtDNA was higher in MHD patients before hemodialysis with the concentration of 129.1±101.3 ng/ml. It was shown that circulatory mtDNA was significantly elevated in MHD patients (P<0.001) (Table 1).

### Circulatory inflammatory markers were elevated in MHD patients

Plasma CRP levels (mg/L) were higher in MHD patients (0.5 to 18.5, median 4.0) than in controls (0.5 to 4.0, median 2.0). Plasma TNF-α levels (pg/ml) were significantly higher in MHD patients (62.5±36.7) than in controls (37.0±2.2) (P = 0.001). Circulatory IL-6 levels (pg/ml) were also evaluated in MHD patients (69.1±53.4) and controls (43.8±8.8), although there was no significant difference between two groups (P = 0.177) (Table 1). It was demonstrated that circulatory inflammatory markers were elevated in MHD patients.

### Circulatory mtDNA was correlated with inflammatory parameters in MHD patients

MHD patients were stratified by the median plasma mtDNA concentration, one group with mtDNA>100 ng/ml, and the other group with mtDNA<100 ng/ml. In the univariate logistic regression analysis, plasma mtDNA was negatively correlated with HGB, serum ALB (P<0.005 for both) and was positively correlated with serum P, product of Ca and P, IL-6 and TNF-α (P<0.05 for all) (Table 2). When these parameters were entered into the multiple logistic regression analysis, TNF-α, product of Ca and P were independently positively associated with plasma mtDNA, while HGB and serum ALB were independently negatively associated with plasma mtDNA (P<0.05 for all) (Table 3). The logistic regression analyses demonstrated that circulatory mtDNA might be associated to the formation of chronic inflammation state in MHD patients.

**Table 2:** Univariate logistic regression analysis—variables associated with plasma mtDNA in MHD patients.

	*P* value	95% CI
Age	0.963	0.955–1.045
Male gender	0.910	0.329–2.640
BMI	0.257	0.927–1.326
SBP	0.438	0.967–1.015
DBP	0.626	0.961–1.069
HD age	0.716	0.991–1.014
DM	0.675	0.325–5.686
HF	0.957	0.234–4.644
Smoking	0.851	0.299–2.710
**HGB**	**0.003**	**0.858–0.968**
**Alb**	**0.002**	**0.606–0.891**
Ca	0.161	0.455–113.227
Alb adjusted Ca	0.061	0.88–288.857
**P**	**0.009**	**1.556–20.551**
**Product of Ca and P**	**0.003**	**1.023–1.120**
CHOL	0.234	0.676–4.956
LDL-C	0.576	0.523–3.210
Ln-Intact-PTH	0.233	0.532–1.166
CRP	0.832	0.207–3.552
**IL-6**	**0.041**	**1.003–1.148**
**TNF-α**	**0.016**	**1.012–1.124**

**Table 3:** Multiple logistic regression analysis—variables independently associated with plasma mtDNA in MHD patients.

	*P* value	95% CI
**TNF-α**	**0.045**	**1.002–1.132**
IL-6	0.131	0.981–1.157
**HGB**	**0.036**	**0.880–0.996**
**ALB**	**0.015**	**0.607–0.949**
Phosphate	0.105	0.751–20.795
**Product of Ca and P**	**0.026**	**1.008–1.137**

### MtDNA induces secretion of inflammatory cytokines in vitro

**Figure 2:**
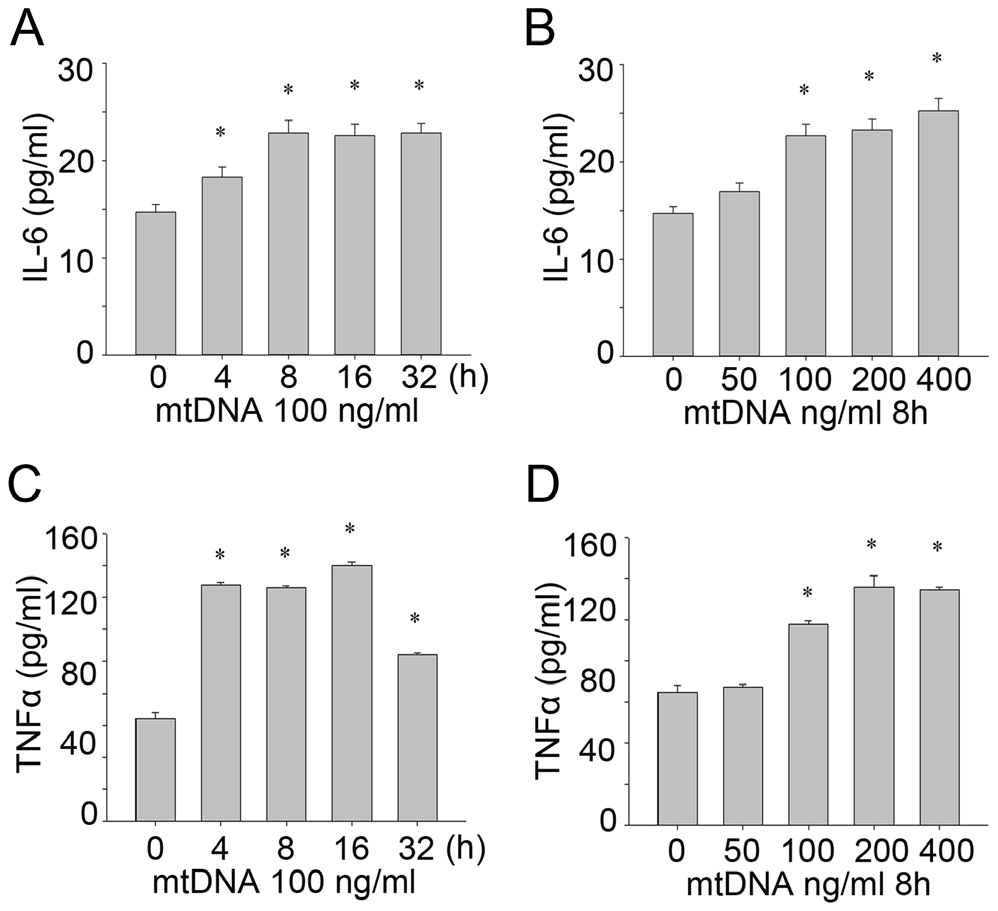
mtDNA induces the cultured THP-1 cells to secrete IL-6 and TNF-α. THP-1 cells (10^6^/ml) were induced to differentiate toward the monocyte/macrophage phenotype by 100 ng/ml of PMA for 36 hours. And then they were incubated with mtDNA for different time and dosage as indicated. (A, B) The effect of mtDNA on release of IL-6 from THP-1. (C, D) The effect of mtDNA on release of TNF-α from THP-1. * P<0.05.

To clarify the relationship between circulatory mtDNA and inflammation, human acute monocytic leukemia cell (THP-1) were treated with mtDNA isolated from human skeletal muscles tissue. For various periods of time at the concentration of 100 ng/ml or different dosage of mtDNA for 8 hours, two typical inflammatory cytokines in the supernatant were measured respectively. The concentration of IL-6 and TNF-α in the supernatant were 14.7±0.8 pg/ml and 64.6±3.4 pg/ml at basal conditions. MtDNA induced IL-6 and TNF-α secretion in a time-dependent manner, starting at a time point as early as 4 hours. The peak values were 22.8±1.3 pg/ml for IL-6 at 8 hours and 159.8±2.6 pg/ml for TNF-α at 16 hours (Fig. 2 A, C). The effect of mtDNA on cytokines secretion was also dose-dependent (Fig. 2 B, D). These results implied that mtDNA could induce secretion of IL-6 and TNF-α from THP-1 cells into the extracellular space, suggesting a potential causal relationship between circulatory mtDNA and inflammation.

### Compared to LF-HD, HF-HD and OL-HDF could partially reduce plasma mtDNA in MHD patients

**Figure 3:**
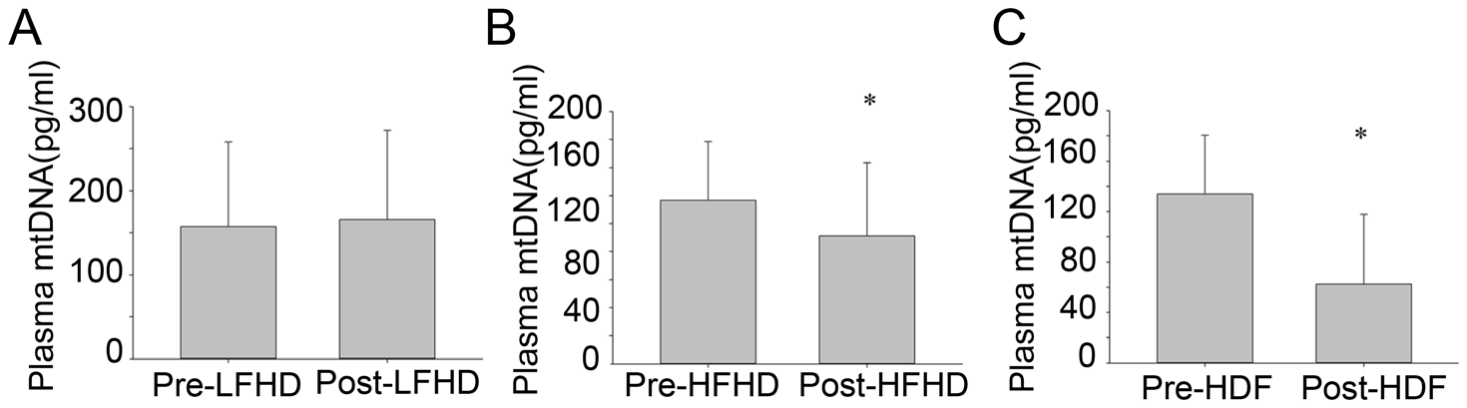
The effects of different dialysis modes on clearance of circulatory mtDNA. A. The effect of LF-HD on plasma mtDNA. B. The effect of HF-HD on plasma mtDNA. C. The effect of OL-HDF on plasma mtDNA. * P<0.05.

The effect of different dialysis patterns, including LF-HD, HF-HD and OL-HDF on the clearance of circulatory mtDNA was evaluated in MHD patients. The abundance of plasma mtDNA before and after a single dialysis was evaluated. There was no difference of the degree of blood concentration and the change of blood pressure among these three groups. In the patients undergoing LF-HD, plasma mtDNA remained unchanged from 157.1±100.1 ng/ml to 165.7±106.1 ng/ml (P = 0.818) (Fig. 3 A). While in patients undergoing HF-HD, plasma mtDNA was decreased from 108.1±40.0 ng/ml to 90.1±53.7 ng/ml (P = 0.023) (Fig. 3 B). Similar results were observed in patients undergoing OL-HDF, plasma mtDNA decreased from 134.0±46.5 ng/ml to 62.6±52.1 ng/ml (P = 0.000) (Fig. 3 C). These data indicated that HF-HD and OL-HDF but not LF-HD could partially reduce plasma mtDNA, which might ameliorate the chronic inflammation state in MHD patients.

### In vitro, HD and HF could fractional remove circulatory mtDNA

**Figure 4:**
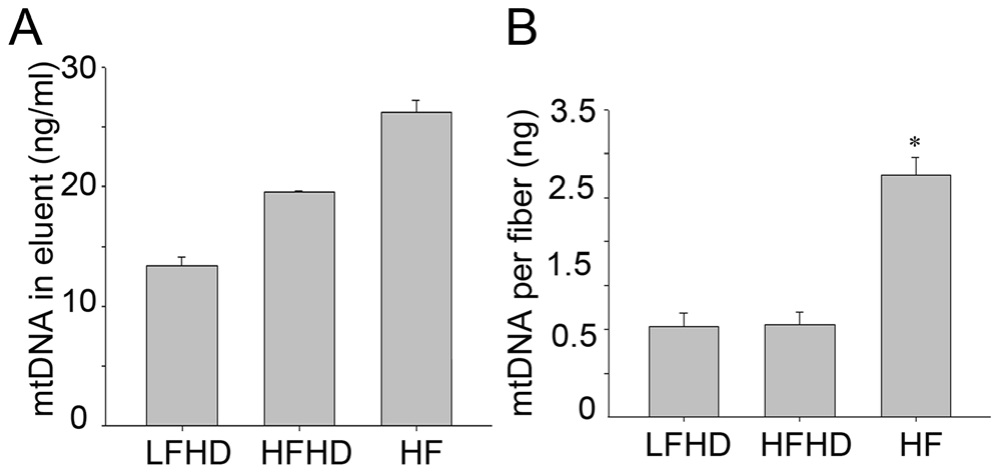
mtDNA absorbed by the dialyzer or filter in the simulated dialysis systems. A. The concentration of mtDNA in eluent of dialyzers. B. The quantity of mtDNA per fiber after soaked overnight by tissue lysis buffer. * P<0.05.

To clarify the mechanism of dialysis modalities on circulatory mtDNA, three simulated dialysis modes were established in vitro. For the LF-HD, combined with the concentration of mtDNA and the final volumes, mtDNA passed through the dialyzer was 5453 ng and remained inside the dialyzer was 2000 ng. For the HF-HD, the amount of mtDNA passed through the dialyzer was 9940 ng and remained inside the dialyzer was 1885 ng. While for the HF, the amount of mtDNA outside the filter was 2002 ng and inside the filter was 3040 ng. Considering the total amount of mtDNA before the treatment was 20000 ng for each dialysis modality, parts of them were absorbed by the dialyzer or filter. mtDNA could be detected in the eluents of the dialyzers and the filter (Fig. 4 A). Following soaking with the eluents, the mtDNA absorbed per fiber could still be detected (Fig. 4 B). It was concluded that mtDNA could partially be removed by LF-HD, HF-HD and HF although quite a number of mtDNA could be absorbed by the dialyzer or filter.

## Discussion

End-stage renal disease (ESRD) is a worldwide public health problem with high morbidity and mortality [20]. Although the long-term survival rate of MHD patients has significantly improved, the mortality of MHD patients was extremely higher than the general population of the same age [21]. Chronic inflammation is highly prevalent in MHD patients, which is a strong independent predictor of all-cause and CVD mortality in those population [1] [22] [23]. It may explain the excess CVD risk in MHD patients, in whom inflammation is common [3]. Certain pro-inflammatory cytokines, such as IL-6 and TNF-α, are considered as early drivers of the inflammatory response. Their elevations in MHD patients are always associated with increased risk of mortality. Common etiologies of inflammation in MHD patients include dialysis-related factors such as the extracorporeal circulation, the quality of the dialysate, the biocompatibility of the dialysis membranes and dialysis catheters as well as non–dialysis-related factors, such as decreased clearance and increased production of cytokines, retention of uremic solutes, increased oxidative stress burden, and high prevalence of co-morbidities associated with inflammation [24].

Circulatory mtDNA is one of the DAMPs reflecting cellular injury, which participating the systemic inflammation in several diseases. Zhang showed that injury released mtDNA into the circulation with functionally important immune consequences. MtDNA could activate human polymorphonuclear neutrophils (PMN) through TLR-9 to induce systemic inflammation [10]. It was also reported recently that mtDNA could act on endothelial cell (EC) and/or PMN via multiple pathways, which could enhance PMN adherence to EC, activate PMN-EC interactions and subsequently increase systemic endothelial permeability [25]. The clinical study implied that plasma mtDNA was an independent indictor with moderate discriminative power to predict the risk of post-traumatic systemic inflammation [26]. Circulatory mtDNA was also elevated in several other diseases such as tumors and sepsis [12], [27], [28]. It has been reported that plasma mtDNA levels could be used as the predictor of outcome in severe sepsis patients in the emergency room [29]. Therefore, circulatory mtDNA might be a pro-inflammatory agent participating systemic inflammation in a variety of diseases.

Given the dependence of renal function on aerobic metabolism, it is not surprising that impairment of normal mitochondrial function—due to insults such as ischaemia, drug toxicity and genetic mitochondrial disease can lead to kidney failure. MtDNA is apt to be exposed to reactive oxygen species (ROS) stress without histone protection. As a result, it is highly susceptible to damage and mutations, with a 10–1,000-fold greater mutation rate than nDNA. Studies have shown significantly increased ROS production, upregulation of COX I and IV expressions, and inactivation of complex IV in peripheral blood mononuclear cells (PBMCs) of patients with phase IV–V chronic kidney disease (CKD), thereby demonstrating the close association between mitochondrial dysfunction and CKD progression [30]. It has been also reported that mtDNA injury was associated with the mortality in MHD patients. The mitochondrial injury markers studied in PBMCs was the mtDNA copy number per cell, and a reduced mitochondrial copy number seems to predict a poor outcome in MHD patients [31]. Although the plasma cf-DNA has been focused recently in MHD patients, the role and mechanisms of circulatory mtDNA in chronic inflammation of MHD patients remains unknown [32].

It was found that circulatory mtDNA was significantly elevated in MHD patients. The mechanism of the elevation of mtDNA in circulation in MHD patients has not been explored. Logistic analysis was done to clarify whether circulatory mtDNA was associated to the chronic inflammatory state in MHD patients. It was demonstrated that plasma mtDNA in MHD patients was positively associated with plasma TNF-α, and was associated with clinical events which have been proven participating in the inflammatory state in MHD patients, such as anemia, hypoproteinemia and hyperphosphatemia [33]. The pro-inflammatory effect of mtDNA was conformed directly in THP-1 cells in vitro. mtDNA could induce the secretion of IL-6 and TNF-α in a time and dose dependent way.

To date, it has been still difficult to ameliorate the chronic inflammatory state in MHD patients. Cytokines such as IL-6 and TNF-α could also not be removed by routine hemodialysis patterns [34]. Considering the pro-inflammatory effect of mtDNA, we further investigated the effect of different routine hemodialysis modes on plasma mtDNA in MHD patients. We found that only HF-HD and OL-HDF but not LF-HD could partially reduce the level of plasma mtDNA. mtDNA in the dialysate could not be detected might due to the large volume of dialysate in single dialysis. Subsequently, in order to further clarify whether mtDNA could go through the dialysis membrane, the simulated dialysis systems in vitro were established. It was demonstrated that mtDNA could be going through the dialysis membrane not only in HF-HD but also in LF-HD. It could also be going through the filer membrane in HF. It was indicated that the form of mtDNA in plasma might be small molecular fragments. This result in vitro was not entirely consistent with the MHD patients undergoing different dialysis modes in vivo. The metabolism of plasma mtDNA might be sophisticated in MHD patients which needs to be explored further.

In conclusion, this is a preliminary clinical study focusing on the circulatory mtDNA on chronic inflammation in MHD patients. It is demonstrated that circulatory mtDNA is elevated in MHD patients. Its level is closely correlated to chronic inflammation in MHD patients. HF-HD and HDF but not LF-HD can partially reduce the circulatory mtDNA. It might be a new marker that provides significant insights into understanding the chronic inflammation of MHD patients and the efficacy of hemodialysis.
